# Validation of an online application to identify potential immune-related adverse events associated with immune checkpoint inhibitors based on the patient’s symptoms

**DOI:** 10.1371/journal.pone.0265230

**Published:** 2022-03-15

**Authors:** Takahiro Osawa, Takashige Abe, Hiroshi Kikuchi, Ryuji Matsumoto, Sachiyo Murai, Takafumi Nakao, Shinji Tanaka, Ayu Watanabe, Nobuo Shinohara

**Affiliations:** 1 Department of Urology, Hokkaido University Graduate School of Medicine, Sapporo, Japan; 2 Pharmacovigilance Division, Ono Pharmaceutical Co., Ltd., Osaka, Japan; University of California, San Francisco, UNITED STATES

## Abstract

**Background:**

Immune checkpoint inhibitors (ICIs) are increasingly being used to treat malignancies. Some patients experience immune-related adverse events (irAEs), which may affect any organ/tissue. IrAEs are occasionally fatal and usually have nonspecific symptoms. We developed a three-step application (https://irae-search.com/) to provide healthcare professionals with information on the diagnosis, treatment options, and published reports for 38 categories of irAEs encountered in clinical practice.

**Methods:**

IrAEs reported in ≥5 cases were identified from articles published between October 2018 and August 2020 by searching Japanese (SELIMIC, JAPIC-Q Service, and JMED Plus) and international (MEDLINE, EMBASE, Derwent Drug File) databases. The cases’ symptoms were entered into the application to identify irAEs, which were verified using the reported diagnosis, to evaluate the application’s sensitivity and specificity.

**Results:**

Overall, 1209 cases (1067 reports) were analyzed. The three most common categories of irAEs were pituitary or adrenal disorders (14% of cases), skin disorders (13%), and diabetes mellitus (10%). The top three primary diseases were lung cancer (364 cases), melanoma (286 cases), and renal cell carcinoma (218 cases). The average sensitivity was 90.8% (range 44.4%–100.0%) initially, and improved to 94.8% (range 83.3%–100.0%) after incorporating the symptoms reported in published cases into the application’s logic for two irAE categories. The average specificity was 79.3% (range 59.1% [thyroid disorders]–98.2% [arthritis]).

**Conclusion:**

irAE Search is an easy-to-use application designed to help healthcare professionals identify potential irAEs in ICI-treated patients in a timely manner to facilitate prompt management/treatment. The application showed high sensitivity and moderate-to-high specificity for detecting irAEs.

## Introduction

Immune checkpoint inhibitors (ICIs) targeting the programmed death-1 (PD-1)/programmed death-ligand 1 (PD-L1) and cytotoxic T-lymphocyte-associated protein 4 (CTLA4) pathways are among the newest entrants into the arsenal for cancer treatment [1]. Although the indications for ICIs vary, they are now available for treating many types of solid cancers and they offer remarkable benefits for patients with advanced diseases [1]. As a consequence, their use is significantly increasing in various oncological settings.

Although ICIs display proven anticancer efficacy, the enhanced immune response that occurs during treatment with these drugs may elicit immune-related adverse events (irAEs) in various body systems and organs [2–16]. Some irAEs are associated with improved overall survival or progression-free survival, and are hence regarded as a marker for the anticancer efficacy of some ICIs [17, 18]. In such cases, continuing treatment for as long as possible is desired from a therapeutic perspective. However, irAEs sometimes become serious, forcing treatment discontinuation, and may even be fatal if not detected and treated in a timely manner. Therefore, irAEs need careful and specialized management. Based on these concepts, early diagnosis and appropriate management of irAEs are vital in order to avoid serious outcomes and to help continuation of ICI therapy.

IrAEs are sometimes identified by members of the healthcare team, and sometimes worked up based on the patient’s symptoms, vital signs, and associated laboratory tests. Recommendations have been developed to aid the diagnosis and appropriate treatment of irAEs [19–22]. Clinical materials have also been introduced to help raise awareness of these events and to improve the diagnostic accuracy in patients treated with ICIs (see for example: [23–32]). However, the materials were often developed for use by specialists in each medical field, rather than by oncology providers in wider clinical practice. Furthermore, the materials are usually organized according to the type of irAE. This means that the healthcare professionals should use the materials with knowledge of the presence and type of irAE in the affected patient. Moreover, the common complaints (e.g., malaise, fatigue, headache), other symptoms, and laboratory test results are often nonspecific and may be misdiagnosed as something other than an irAE. In these circumstances, the patient may receive symptomatic or stop-gap therapies rather than a systemic treatment targeting the underlying cause. Therefore, there was an unmet need for developing an easy-to-use application to help healthcare professionals quickly identify potential irAEs based on the patient’s subjective symptoms, laboratory tests, or vital signs. In this context, we developed a three-step online application (https://irae-search.com/) that was designed to easily identify over 500 potential irAEs that may be encountered in clinical practice.

In this study, we performed a literature review of published cases of irAEs and used the application to evaluate the irAEs based on the symptoms reported in each case. We report the results of the literature search, and the sensitivity and specificity of the application for detecting 38 categories of irAEs. We also report the improvement in the application’s sensitivity that was achieved by revising the links in the application between symptoms and irAEs with low initial sensitivity.

## Methods

### Online application

A newly developed web application was used in this study. The application was developed by a software provider (3H Clinical Trial Corporation) in collaboration with the authors. The logic linking the symptoms and irAEs was developed and refined as follows. First, over 500 irAEs were extracted from specialized books of medical diagnosis [33, 34], Japanese Society of Medical Oncology Cancer Immunotherapy Guidelines [22], as well as the Package Inserts, Patient Guides, and Proper Use Guides for ICIs, including those for nivolumab and nivolumab–ipilimumab [35, 36]. Next, the irAEs were classified into 38 categories (Table 1 and S1 Table). Finally, the symptoms were listed for each irAE, and graded into three classes by specificity (highly specific symptoms for a particular irAE; frequently observed symptoms for a particular AE; and other symptoms that occur in many types of irAEs).

**Table 1:** Immune-related adverse events included in the online application.

Category
Arrhythmia
Arthritis
Cardiac disorders including myocarditis
Cholangitis
Cystitis/urethritis
Cytokine release syndrome
Diabetes mellitus
Thyroid disorders
Encephalitis/meningitis etc
Gastrointestinal bleeding
Gastrointestinal perforation
Gastroesophageal reflux
Hepatic encephalopathy
Hepatitis/hepatic disorders
Ileus
Infusion reactions
Interstitial pneumonia and other lung disorders
Irritable bowel syndrome
Lower gastrointestinal disorders
Myositis/myopathy
Nephritis/renal disorders
Neuropathy
Oral disorders
Pancreatitis
Peritonitis
Pituitary or adrenal disorders
Pneumothorax/pleural effusion
Pulmonary/respiratory tract haemorrhage
Sarcoidosis
Serious haematologic disorders
Sjögren’s syndrome
SJS, TEN, pemphigoid, erythema multiforme, and other skin disorders
Thrombosis/embolism
Upper gastrointestinal disorders
Uveitis, VKH disease, iridocyclitis, and other ophthalmologic disorders
Vasculitis
Vertigo
VKH disease

The diagnoses associated with each irAE category are listed in S1 Table.

SJS, Stevens–Johnson syndrome; TEN, toxic epidermal necrolysis; VKH, Vogt–Koyanagi–Harada.

**Fig 1:**
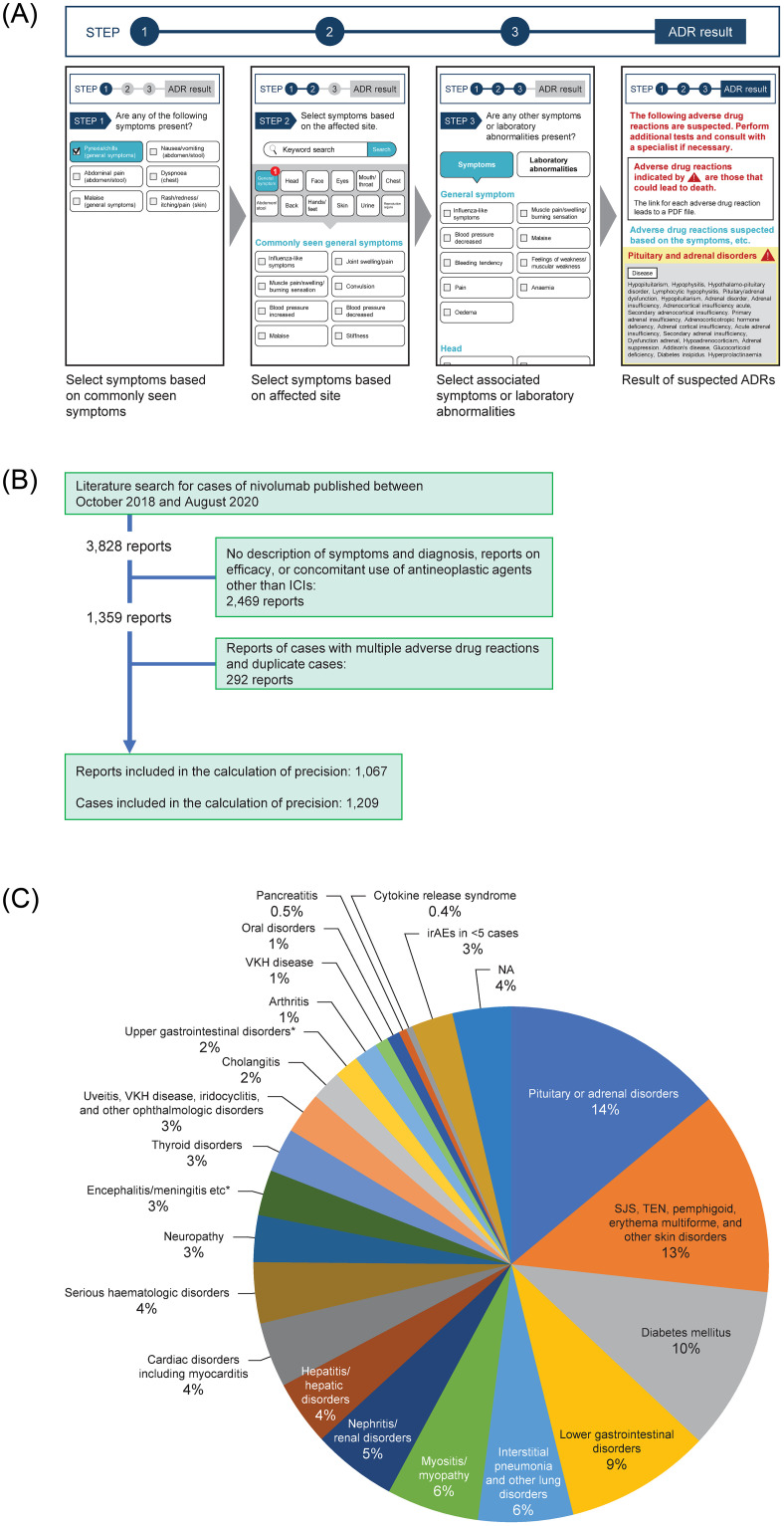
(A) Schematic overview of the three steps of the application for identifying potential immune-related adverse events (irAEs) based on symptoms, laboratory tests, and vital signs. (B) Literature flow chart. (C) Frequencies of irAEs. NA (not applicable) indicates events that could not be classified into the 38 categories of irAEs included in the application. See Table 1 and S1 Table for the list of irAEs and associated diagnoses included in the application.

The application involves three steps: (1) Select symptoms from choices of common symptoms, (2) Select symptoms by body parts (ambiguous searches are possible), and (3) Select related symptoms and abnormal laboratory tests, which are displayed based on the selections made in steps 1 and 2 (Fig 1A). When a user selects a highly specific or a symptom common to a particular irAE, the application is designed to display the strongly suspected irAE in the final results screen. If the user only selects ‘other’ symptoms, the suspected irAE is only displayed if all of the ‘other’ selected symptoms are commonly linked to the particular irAE. A network map illustrating the links between the symptoms and irAEs is presented in S1 File.

The application frontside uses a combination of HyperText Markup Language, cascading style sheets, and JavaScript running on a Java SE Development Kit 16.0.1 server and a Java-based Spring Boot framework. Data are stored in a PostgreSQL pgAdmin4 database running on Linux.

### Literature search for application validation

In order to validate the application, we assessed its sensitivity and specificity by performing a comprehensive literature search of Japanese (SELIMIC, JAPIC-Q Service, and JMED Plus) and international (MEDLINE, EMBASE, and Derwent Drug File) databases. The search strategies were developed specifically for each database to identify case-reports describing nivolumab (Nivolumab, Opdivo, ONO-4538, BMS-936558, MDX-1106) and adverse events (adverse reactions, adverse events, toxicity, poisoning, safety, drug interactions, carcinogenicity, teratogenicity) published between October 2018 and August 2020. The published abstracts were retrieved. Articles were discarded if they did not describe the symptoms or a diagnosis, reported efficacy only, involved concomitant use with antineoplastic drugs other than an ICI, or described multiple adverse drug reactions. The literature search was conducted by researchers at Ono Pharmaceutical Co., Ltd., and data were extracted from each case-report in a retrospective manner.

### Ethics

Ethical approval and consent were not required for the literature searches reported in this article, or for developing the application because it does not record any patient information in a database. Search logs are reviewed for the purpose of system improvements.

### Data analysis

Patient characteristics, symptoms, and adverse drug reactions (including the type of irAE) were extracted from each abstract, tabulated in a database, and analyzed descriptively to determine the patient/disease characteristics by researchers at Ono Pharmaceutical Co., Ltd. in a non-blinded manner. The retrieved information was then used to determine the sensitivity and specificity of the application. The sensitivity was calculated as the rate at which a particular irAE was determined by this application to be an irAE that is finally diagnosed by the physician (i.e., the proportion of true positives that are correctly identified by the application) and the specificity was calculated as the rate of nonspecific irAEs (i.e., proportion of true negatives that are correctly identified) (S2 Table). After performing these analyses, it was discovered that some irAEs had a low sensitivity. Therefore, the links between symptoms and irAEs were reviewed and refined based on the information published in each case and clinical experience. As a consequence of the literature review, several new symptoms were added to the application. The sensitivity and specificity were then recalculated using the same cases. Microsoft Excel (Microsoft Corp., Redmond, WA, USA) was used for data aggregation and all analyses. The network map was visualized using the Compound Spring Embedder (CoSE) layout in Cytoscape 3.8.2 (https://cytoscape.org/) [37].

## Results

### Literature review

The literature search initially retrieved 3828 published reports, of which 1067 were included in the analyses (Fig 1B). These reports included 1209 cases treated with nivolumab or another ICI. The data extracted for these cases are presented in S3 Table. The cases included 835 men and 337 women (not recorded for 37 cases) (Table 2), with a mean age of 65.0 years (range 9–93 years; available in 1063 cases). The three most frequent primary diseases were lung cancer (364 cases), melanoma (286 cases), and renal cell carcinoma (218 cases). Nivolumab (988 cases), nivolumab plus ipilimumab (190 cases), pembrolizumab (63 cases), and ipilimumab (44 cases) were the four most frequently used treatments.

**Table 2:** Overview of the cases.

Characteristic	Number of cases^a^
Number of cases	1209
Sex, *n* (%)	
Men	835 (69.1%)
Women	337 (27.9%)
Not reported/not available	37 (3.1%)
Age, years	
Mean ± SD (range)	65.0 ± 12.0 (9–93), *n* = 1063
<65 years, *n* (%)	449 (37.1%)
≥65 years, *n* (%)	614 (50.8%)
Age unknown/not precisely recorded, *n* (%)	146 (12.1%)
Primary disease^b^, *n*	
Lung cancer	364
Melanoma	286
Renal cell carcinoma	218
Gastric cancer	126
Head and neck cancer	73
Hodgkin lymphoma	20
Malignant pleural mesothelioma	15
Esophageal cancer	11
Others	66
Unknown	38
Suspected drug^b^, *n*	
Nivolumab	988
Nivolumab + ipilimumab	190
Pembrolizumab	63
Ipilimumab	44
Atezolizumab	5
Durvalumab + tremelimumab	2
Unknown	2

^a^Values are number of cases, unless specified otherwise.

^b^Because some cases had multiple primary diseases or received multiple treatments, the numbers may exceed the total number of cases retrieved in the literature search.

### Verification of the application

Fig 1C shows the relative frequencies of irAEs reported in ≥5 cases; this figure includes >95% of cases in which irAEs were identified. IrAEs that occurred in more than ≥5% of cases were pituitary or adrenal disorders (14% of cases); SJS, TEN, pemphigoid, erythema multiforme, and other skin disorders (13%); diabetes mellitus (10%); lower gastrointestinal disorders (9%); interstitial pneumonia and other lung disorders (6%); myositis/myopathy (6%); and nephritis/renal disorders (5%).

The sensitivity and specificity of the application are shown in Table 3, which includes 21 categories of irAEs reported in ≥5 cases. The sensitivity ranged from 44.4% for upper gastrointestinal disorders to 100.0% for pituitary or adrenal disorders, lower gastrointestinal disorders, cholangitis, Vogt–Koyanagi–Harada disease, oral disorders, and cytokine release syndrome. The average sensitivity was 83.3%. The sensitivity was <80% for two categories, namely encephalitis/meningitis etc (51.4%) and upper gastrointestinal disorders (44.4%). The specificity ranged from 59.1% (thyroid disorders) to 98.2% (arthritis), with an average of 79.3%. The specificity was <80% for eight categories: pituitary or adrenal disorders; lower gastrointestinal disorders; myositis/myopathy; nephritis/renal disorders; hepatitis/hepatic disorders; cardiac disorders including myocarditis; serious haematologic disorders; and thyroid disorders.

**Table 3:** Sensitivity and specificity of the application for predicting irAEs.

irAE category	Cases, *n*	Sensitivity (%)	Specificity (%)
Pituitary or adrenal disorders	171	100.0	59.9
SJS, TEN, pemphigoid, erythema multiforme, and other skin disorders	157	89.8	83.7
Diabetes mellitus	126	98.4	89.5
Lower gastrointestinal disorders	112	100.0	74.7
Interstitial pneumonia and other lung disorders	73	94.5	82.6
Myositis/myopathy	71	95.8	72.4
Nephritis/renal disorders	65	93.8	70.2
Hepatitis/hepatic disorders	50	94.0	62.3
Cardiac disorders including myocarditis	50	94.0	64.1
Serious haematologic disorders	47	97.9	67.0
Neuropathy	36	83.3	84.6
Encephalitis/meningitis etc^a^	35	51.4 → 85.7	91.0 → 87.6
Thyroid disorders	34	94.1	59.1
Uveitis, VKH disease, iridocyclitis, and other ophthalmologic disorders	32	96.9	94.6
Cholangitis	23	100.0	83.6
Upper gastrointestinal disorders^a^	19	44.4 → 94.7	88.3 → 83.9
Arthritis	18	94.4	98.2
VKH disease	10	100.0	89.0
Oral disorders	10	100.0	92.4
Pancreatitis	6	83.3	85.4
Cytokine release syndrome	5	100.0	80.1
Average value^b^	-	90.8 → 94.8	79.7 → 79.3

^a^The sensitivity and specificity are shown before and after refining the underlying logic for these irAE categories.

^b^The sensitivity and specificity (with averages) were calculated for irAE categories with ≥5 cases.

irAE, immune-related adverse events; SJS, Stevens–Johnson syndrome; TEN, toxic epidermal necrolysis; VKH, Vogt–Koyanagi–Harada.

### Refinement of the application for irAEs with low sensitivity

Because the application’s sensitivity was relatively low (<80%) for upper gastrointestinal disorders and encephalitis/meningitis etc, we revised the links between symptoms and irAEs to improve the application’s sensitivity for predicting these categories of irAEs.

S4 Table summarizes the diagnoses and symptoms in reported cases with upper gastrointestinal disorders. Based on the literature search, we identified several common symptoms, including weight decreased (6 of 19 cases), dysphagia (5 of 19 cases), and anorexia (5 of 19 cases), which were originally included in the application but were not linked to upper gastrointestinal disorders. Adding links between these symptoms and this irAE to the application increased the sensitivity from 44.4% to 94.7% (S1 Fig). The specificity remained high (before: 88.3%; after: 83.9%) (Table 3).

Similarly, for encephalitis/meningitis etc, we identified several relatively common symptoms that were not originally linked to this irAE category or were not included as possible symptoms in the application (S5 Table). Dyslalia (2 of 35 cases) was replaced with language disorders (6 of 35 cases), and new links were added for memory impairment (2 of 35 cases) and feeling of weakness/muscular weakness (6 of 35 cases) (S2 Fig). These changes increased the sensitivity for encephalitis/meningitis etc from 51.4% to 85.7%. The specificity remained high (before: 91.0%; after: 87.6%) (Table 3).

The average sensitivity and specificity after implementing these changes were 94.8% and 79.3%, respectively.

## Discussion

An online application (irAE Search) was developed with the aim of providing healthcare professionals with an easy-to-use search application to help the user identify potential irAEs based on a combination of symptoms, laboratory tests, and vital signs in the affected patient. It is intended for use by healthcare professionals, including those who are relatively inexperienced in using ICIs or their associated risks. Upon entering the information in three simple steps, the application provides the user with drug use guides and case reports relevant to the most probable irAEs. It has also provides links to many external references and clinically relevant resources. The healthcare professionals can access the application via a web-browser on a computer or via a smartphone/tablet.

In this study, we first verified the irAE categories by performing a systematic literature review, which retrieved 1209 cases. Among irAEs observed in ≥5 cases, the application had a sensitivity reaching 100.0% for several categories of irAEs (pituitary or adrenal disorders, lower gastrointestinal disorders, cholangitis, Vogt–Koyanagi–Harada disease, and oral disorders) Based on these findings, the application is expected to predict all true-positive cases for these categories. However, the application showed relatively low sensitivities for upper gastrointestinal disorders (44.4%) and encephalitis/meningitis etc (51.4%). Revising the links between symptoms and irAEs in the application using the information from the literature review increased the sensitivities for both categories (upper gastrointestinal tract disorders: from 44.4% to 94.7%; encephalitis/meningitis etc: from 54.1% to 85.7%). Finally, the sensitivity improved to >80% for all irAE categories, with an average of 94.8%.

The specificity of the application ranged from 59.1% (thyroid disorder) to 98.2% (arthritis), with an average of 79.3%. Because the specificity represents the accuracy of detecting a true negative case, there is a risk of incorrectly predicting irAEs with low specificity, such as endocrine disorders (59.8% for pituitary or adrenal disorders, 59.1% for thyroid disorders). Furthermore, some symptoms may overlap multiple categories of irAEs. For example, fatigue and anorexia were observed in cases with endocrine disorders. However, they may also occur in cases with other irAEs and are features of the general malaise of cancer patients [38]. For this reason, the specificity was relatively low for some irAEs. Generally, purely diagnostic tests/assays are expected to show high specificity to avoid incorrect diagnoses. However, our application is not intended to be used for purely diagnostic purposes. Rather, it is designed to raise suspicion of potential irAEs and prompt the physician to perform additional tests to help confirm the irAE and to consider appropriate interventions. The probable diagnoses are ordered (descending order) based on the weight applied to the selected symptoms in the application, which also highlights potentially fatal irAEs. Therefore, the low specificity for some irAEs is not necessarily a limitation of the application. In fact, the low specificity may be unavoidable to ensure high sensitivity and avoid overlooking irAEs in clinical practice.

The indications for ICIs are steadily increasing, particularly dual ICI therapy (e.g., nivolumab plus ipilimumab) [39] and their use is expected to expand beyond highly specialized centers. Healthcare professionals outside these centers may be less aware of the risks or signs of irAEs, and they are likely to benefit most from a simple application, such as this one. Thus, this application is valuable for helping healthcare professionals investigate suspected irAEs in patients treated with ICIs.

### Limitations

There are some limitations of the application and the present study to discuss. The application is designed to identify potential AEs of ICIs and cannot be applied to AEs caused by other therapeutic reagents. For example, AEs specifically related to tyrosine kinase inhibitors cannot be appropriately identified in patients with renal cell cancer receiving tyrosine kinase inhibitor–ICI combination therapy, a regimen that will be used more frequently in the near future. Because the application is not embedded within electronic medical record systems, healthcare professionals need to be aware of the application to ensure they can access and use it. It is currently only available in Japanese, although an English version is under consideration. For this study, we searched the medical literature using terms to identify nivolumab-treated cases. However, these terms retrieved some cases treated with other ICIs (with or without nivolumab), but these cases were included in the analyses. Cases with concurrent irAEs and cases who received concomitant cytotoxic anticancer therapy were excluded from the study. Because we did not evaluate the sensitivity or specificity for irAEs that occurred in <5 cases, caution should be exercised in such cases. Additionally, because case reports often describe rare diseases/disorders, it is possible that our search overestimated the prevalence of rare disorders or symptoms. Another possible limitation is that the data were extracted in a retrospective manner from published case reports by researchers who were aware of the final diagnosis; these factors may influence the sensitivity or specificity. This study did not investigate the feasibility or utility of the application by healthcare professionals in clinical practice. Future studies, including surveys of the users, may provide insight into its use and opportunities for further development.

## Conclusions

In conclusion, irAE Search is an easy-to-use only application that was designed to help healthcare professionals identify potential irAEs in patients treated with ICIs in a timely manner and to facilitate prompt management/treatment. The application showed high sensitivity and moderate-to-high specificity for detecting irAEs in clinical practice. For two categories of irAEs, the sensitivity was improved by incorporating information about symptoms from the published cases. Ongoing development of the application is expected to yield further improvements in the sensitivity and specificity for identifying potential irAEs.

## Supporting information

S1 Table Immune-related adverse events and associated diagnoses included in the online application. (PDF)

S2 Table Assessment of sensitivity and specificity. (PDF)

S3 Table Examples of cases of immune-related adverse events identified by the literature search. (PDF)

S4 Table Diagnoses and symptoms observed in 19 cases with upper gastrointestinal disorders. (PDF)

S5 Table Diagnoses and symptoms observed in 35 cases with encephalitis/meningitis etc. (PDF)

S1 Fig Uplift in sensitivity for predicting upper gastrointestinal disorders after incorporating links to common symptoms described in 19 cases retrieved by the literature search. (PDF)

S2 Fig Uplift in sensitivity for predicting encephalitis/meningitis etc after incorporating links to common symptoms described in 35 cases retrieved by the literature search. (PDF)

S1 File Network map illustrating the links between the symptoms and irAEs. The symptoms and irAEs are linked using red or grey lines. Red lines indicate high specificity of a symptom for a particular irAE, grey lines indicate low specificity of a symptom to each linked irAE. Symptoms are colored blue, where the intensity of color represents the number of irAEs the symptom is connected to. Light blue symptoms are linked to one or a few irAEs, and are classified as highly specific symptoms. By contrast, dark blue (e.g., pyrexia/chills, nausea/vomiting, and abdominal pain) indicates broad symptoms, which are linked to many irAEs.
(PDF)

S2 File Complete list of cases retrieved by the literature search. (XLSX)
