# Supplementary material for: Validation of an online application to identify potential immune-related adverse events associated with immune checkpoint inhibitors based on the patient’s symptoms
Source: PLoS One. 2022 Mar 15;17(3):e0265230. doi: 10.1371/journal.pone.0265230 (PMC8923505; doi:10.1371/journal.pone.0265230)
Supplement: S1 Table — (PDF) [file pone.0265230.s001.pdf]

S1 Table. Immune-related adverse events and associated diagnoses included in the online application.

irAE category	Associated diagnoses
Arrhythmia	Arrhythmia, atrial fibrillation, atrial flutter, sinus bradycardia, sinus tachycardia, supraventricular extrasystoles, supraventricular tachycardia, tachyarrhythmia, ventricular arrhythmia, ventricular extrasystoles
Arthritis	Arthritis, autoimmune arthritis, chondrocalcinosis, polyarthritis, seronegative arthritis, synovial cyst, synovitis
Cardiac disorders including myocarditis	Cardiac failure, cardiac failure acute, cardiac failure high output, cardiac hypertrophy, cardiomegaly, ischaemic heart disease, myocardial infarction, myocarditis, orthostatic hypotension
Cholangitis	Cholangitis, cholangitis sclerosing
Cystitis/urethritis	Allergic cystitis, cystitis, cystitis erosive, cystitis glandularis, cystitis haemorrhagic, cystitis interstitial, cystitis noninfective, cystitis ulcerative, cystitis-like symptom, eosinophilic cystitis, lupus cystitis, pyelocystitis, urethritis
Cytokine release syndrome	Cytokine release syndrome
Diabetes mellitus	Diabetes mellitus, diabetic ketoacidosis, fulminant type 1 diabetes mellitus, insulin deficiency diabetes, type 1 diabetes mellitus, type 2 diabetes mellitus
Thyroid disorders	Autoimmune hypothyroidism, autoimmune thyroiditis, Basedow’s disease, dysfunction thyroid, hyperthyroidism, hypothyroidism, primary hypothyroidism, secondary hypothyroidism, silent thyroiditis, thyroid disorder, thyroiditis, thyroiditis acute, thyroiditis chronic, thyroiditis subacute, thyrotoxicosis

**irAE category**	**Associated diagnoses**
Encephalitis/ meningitis etc	Arachnoiditis, brain oedema, cranial nerve disorder, encephalitis, encephalitis autoimmune, limbic encephalitis, meningism, meningitis, meningitis aseptic, meningitis noninfective, myelitis, Parkinson's disease, subdural haematoma, VIth nerve paralysis
Gastrointestinal bleeding	Duodenitis haemorrhagic, enterocolitis haemorrhagic, gastric haemorrhage, gastritis haemorrhagic, gastroduodenal haemorrhage, gastrointestinal haemorrhage, intestinal haemorrhage, large intestinal haemorrhage, lower gastrointestinal haemorrhage, proctitis haemorrhagic, small intestinal haemorrhage, upper gastrointestinal haemorrhage
Gastrointestinal perforation	Bowel perforation, diverticular perforation, duodenal perforation, gastric perforation, gastrointestinal perforation, ileal perforation, intestinal perforation, jejunal perforation, large intestine perforation, lower gastrointestinal perforation, perforation, small intestine perforation
Gastroesophageal reflux	Gastroesophageal reflux, gastroesophageal reflux disease,
Hepatic encephalopathy	Coma hepatic, hepatic encephalopathy,
Hepatitis/hepatic disorders	Acute hepatic failure, acute on chronic liver failure, autoimmune hepatitis, chronic hepatic failure, drug-induced liver injury, drug-induced liver injury, fulminant hepatic failure, hepatic failure, hepatic function disorder, hepatitis, hepatitis acute, Immune-mediated hepatitis, liver disorder, subacute hepatic failure
Ileus	Duodenal obstruction, ileus, ileus paralytic, ileus spastic, intestinal obstruction, large intestinal obstruction, mechanical ileus, small intestinal obstruction, subileus

**irAE category**	**Associated diagnoses**
Infusion reactions	Anaphylactic reaction, anaphylactoid reaction, anaphylaxis, drug hypersensitivity, hypersensitivity, infusion reaction, infusion related reaction, type I hypersensitivity
Interstitial pneumonia and other lung disorders	Acute interstitial pneumonitis, acute pulmonary oedema, acute respiratory distress syndrome, acute respiratory failure, bronchiolitis, bronchitis, diffuse alveolar damage, interstitial pneumonia, lung disorder, lung infiltration, mediastinitis, organizing pneumonia, pneumonia, pneumonia aspiration, pneumonitis, pulmonary oedema, pulmonary tuberculosis, radiation pneumonitis, respiratory failure, tracheobronchitis, upper respiratory tract inflammation
Irritable bowel syndrome	Irritable bowel syndrome
Lower gastrointestinal disorders	Appendicitis perforated, autoimmune colitis, colitis, colitis ischaemic, colitis microscopic, colitis ulcerative, diverticulitis, enteritis, enterocolitis, gastroenteropathy, gastrointestinal inflammation, immune-mediated enterocolitis, large intestinal ulcer, necrotizing colitis, proctitis
Myositis/myopathy	Asthenia, autoimmune myositis, muscle rigidity, muscle spasms, musculoskeletal stiffness, myasthenia gravis, myasthenic syndrome, myopathy, myositis, polymyalgia rheumatica, polymyositis, rhabdomyolysis
Nephritis/renal disorders	Acute kidney injury, acute renal failure, autoimmune glomerulonephritis, autoimmune nephritis, glomerulonephritis, glomerulonephritis membranous, glomerulonephritis minimal lesion, interstitial renal impairment, nephritis, nephritis interstitial, pyelonephritis, pyelonephritis acute, renal disorder, renal failure, renal impairment, renal tubular acidosis, tubulointerstitial nephritis

irAE category	Associated diagnoses
Neuropathy	Acute motor-sensory axonal neuropathy, autoimmune neuropathy, autonomic neuropathy, axonal neuropathy, chronic inflammatory demyelinating polyneuropathy, demyelinating disorders, demyelinating polyneuropathy, demyelinating polyneuropathy, demyelination, facial nerve disorders, fine motor skill dysfunction, Guillain-Barre syndrome, Miller Fisher syndrome, mononeuropathy multiplex, nerve disorders, neuralgia, oral dysesthesia, peripheral motor neuropathy, peripheral nerve disorder, peripheral neuropathy, peripheral sensorimotor neuropathy, peripheral sensory neuropathy, polyneuropathy, trigeminal neuralgia
Oral disorders	Aphthous ulcer, oral disorder, stomatitis
Pancreatitis	Autoimmune pancreatitis, pancreatitis, pancreatitis acute
Peritonitis	Peritonitis
Pituitary or adrenal disorders	Acute adrenal insufficiency, Addison's disease, adrenal cortical insufficiency, adrenal disorder, adrenal insufficiency, adrenal suppression, adrenocortical insufficiency acute, adrenocorticotropic hormone deficiency, diabetes insipidus, dysfunction adrenal, glucocorticoid deficiency, hyperprolactinemia, hypoadrenocorticism, hypophysitis, hypopituitarism, hypothalamo-pituitary disorder, lymphocytic hypophysitis, pituitarism, pituitary failure, primary adrenal insufficiency, secondary adrenal insufficiency, secondary adrenocortical insufficiency
Pneumothorax/ pleural effusion	Pleural effusion, pneumothorax,
Pulmonary/ respiratory tract haemorrhage	Pulmonary haemorrhage, respiratory tract haemorrhage
Sarcoidosis	Heerfordt’s syndrome, sarcoidosis,

irAE category	Associated diagnoses
Serious haematologic disorders	Agranulocytosis, alkalosis, anaemia, aplasia pure red cell, autoimmune haemolytic anaemia, autoimmune neutropenia, cold type haemolytic anaemia, eosinopaenia, febrile neutropaenia, haemolysis, haemolytic anaemia, haemophagocytic lymphohistiocytosis, haemophagocytic syndrome, haemophilia A, iron deficiency anaemia, leukocytosis, leukopenia, lymphopenia, neutropaenia, normochromic normocytic anaemia, serious blood disorder, thrombocytopaenia, thrombocytopenic purpura
Sjogren's syndrome	Sjogren's syndrome
SJS, TEN, pemphigoid, erythema multiforme, and other skin disorders	Autoimmune dermatitis, cellulitis, dermatitis, dermatitis acneiform, dermatitis allergic, dermatitis bullous, dermatitis exfoliative, dermatitis exfoliative generalized, dermatitis psoriasiform, eczema asteatotic, eczema nummular, erysipelas, erythema annulare, exfoliative rash, fixed eruption, folliculitis, guttate psoriasis, herpes zoster, keratosis pilaris, leukoplakia, lichenoid keratosis, neurodermatitis, oculomucocutaneous syndrome, palmar-plantar erythrodysaesthesia syndrome, pemphigoid, photosensitivity reaction, pruritus, psoriasis, pustular psoriasis, rosacea, seborrheic dermatitis, skin disorder, skin hyperpigmentation, skin hypopigmentation, skin mass, skin ulcer, Stevens–Johnson syndrome (SJS), toxic epidermal necrolysis (TEN), toxic skin eruption, vitiligo
Thrombosis/ embolism	Carotid artery thrombosis, deep vein thrombosis, embolism, embolism venous, pulmonary embolism, pulmonary venous thrombosis, subclavian vein thrombosis, venous thromboembolism, venous thrombosis, venous thrombosis limb

irAE category	Associated diagnoses
Upper gastrointestinal disorders	Chronic gastritis, duodenal ulcer, duodenal ulcer, duodenitis, erosive duodenitis, gastric ulcer, gastritis, gastritis erosive, gastroenteropathy, gastrointestinal inflammation, oesophagitis, ulcerative duodenitis
Uveitis, VKH disease, iridocyclitis, and other ophthalmologic disorders	Autoimmune uveitis, conjunctival oedema, conjunctivitis, corneal disorder, episcleritis, eye pruritus, iridocyclitis, iritis, ocular pemphigoid, optic neuritis, scleritis, ulcerative keratitis, uveitis, vitreous floaters, vitreous haemorrhage, VKH disease
Vasculitis	Angiopathy, hypersensitivity vasculitis, phlebitis, temporal arteritis, vasculitis
Vertigo	Vertigo
VKH disease	VKH disease

VKH, Vogt–Koyanagi–Harada
