# Supplementary material for: Validation of an online application to identify potential immune-related adverse events associated with immune checkpoint inhibitors based on the patient’s symptoms
Source: PLoS One. 2022 Mar 15;17(3):e0265230. doi: 10.1371/journal.pone.0265230 (PMC8923505; doi:10.1371/journal.pone.0265230)
Supplement: S2 Table — (PDF) [file pone.0265230.s002.pdf]

S2 Table. Assessment of sensitivity and specificity.

		irAE predicted by the online application	
		A	other than A
irAE described in the abstract	A	a	b
	Other than A	c	d

Sensitivity: rate at which a particular irAE is determined to be that particular irAE = *a*/*a*+*b*

Specificity: rate of non-specific irAEs = *d*/*c*+*d*
