# Supplementary material for: Validation of an online application to identify potential immune-related adverse events associated with immune checkpoint inhibitors based on the patient’s symptoms
Source: PLoS One. 2022 Mar 15;17(3):e0265230. doi: 10.1371/journal.pone.0265230 (PMC8923505; doi:10.1371/journal.pone.0265230)
Supplement: S3 Table — (PDF) [file pone.0265230.s003.pdf]

S3 Table. Examples of Cases of immune-related adverse events identified by the literature search.

Citation	Pubmed ID	Age	Sex	Primary disease	Suspected drug	Symptoms and abnormal laboratory values	Diagnosis
Chang A, et al. Myocarditis with radiotherapy and immunotherapy in multiple myeloma. Journal of Oncology Practice. 2018;14(9):561-564	30004825	47	W	Refractory myeloma	Nivolumab	Chest pain, pyrexia	Acute autoimmune myocarditis
Kopecky J, et al. Nivolumab-induced encephalopathy in a man with metastatic renal cell cancer: A case report. Journal of Medical Case Reports. 2018;12(1):262	30217214	63	M	Renal cell carcinoma	Nivolumab	Behavioral changes, uncontrollable motion	Encephalitis
Joel TB, et al. Scleroderma-like skin changes induced by checkpoint inhibitor therapy. American Journal of Dermatopathology. 2018;40(6):e92	N/A	61	M	Oligometastatic renal cell carcinoma	Nivolumab	Skin hypertrophy, abdominal wall oedema with downward progression to the trunk and lower extremities	Scleroderma
Usui Y, et al. Mechanistic analysis of cytokine release syndrome after autologous HSCT following PD-1 blockade. Clinical Blood. 2018.1;59(9):OS2-5A-3	33512708	63	M	Hodgkin’s lymphoma	Nivolumab	Erythema, hypotension, hypoxia, kidney function decreased, high fever	Cytokine release syndrome

For the full list of cases, please refer to the accompanying Excel file online (S2 File).
