# Supplementary material for: Validation of an online application to identify potential immune-related adverse events associated with immune checkpoint inhibitors based on the patient’s symptoms
Source: PLoS One. 2022 Mar 15;17(3):e0265230. doi: 10.1371/journal.pone.0265230 (PMC8923505; doi:10.1371/journal.pone.0265230)
Supplement: S4 Table — (PDF) [file pone.0265230.s004.pdf]

S4 Table. Diagnoses and symptoms observed in 19 cases with upper gastrointestinal disorders.

Diagnosis	Symptoms
Duodenitis	Nausea/vomiting, anorexia, abdominal pain
Fistula between upper trachea and oesophagus	Dysphagia, dyspnoea
Nivolumab-induced gastritis	Anorexia
Gastritis	Weight decreased, nausea/vomiting, abdominal pain
Active oesophagitis	Dysphagia, sore throat/itching throat
Duodenitis/enterocolitis	Diarrhoea/faeces soft, abdominal pain
Oesophagitis ulcerative	Dysphagia, sore throat/itching throat
Autoimmune gastritis	Weight decreased, nausea/vomiting, abdominal pain, CRP increased, lipase increased
Subacute gastritis	Weight decreased, nausea/vomiting, abdominal pain
Acute haemorrhagic gastritis	Nausea/vomiting, abdominal pain
Gastroduodenitis haemorrhagic	Blood pressure decreased, nausea/vomiting, abdominal pain, lipase increased
Protein-losing gastroenteropathy	Oedema, diarrhoea/faeces soft, anorexia
Autoimmune mucositis/esophagitis	Malaise, weight decreased, dysphagia
Active gastric and duodenal inflammation	Weight decreased, dehydration, nausea/vomiting, abdominal pain
Stenosis of abdominal oesophagus	Nausea/vomiting, anorexia
Gastrointestinal disorder	Dysphagia, labial/oral erosion, sore throat/itching throat
Subacute gastritis	Weight decreased, nausea/vomiting
Gastritis	Anorexia, abdominal distension/discomfort
Diffuse haemorrhagic gastroenteritis	Abdominal pain

CRP, C-reactive protein
