# Supplementary material for: Validation of an online application to identify potential immune-related adverse events associated with immune checkpoint inhibitors based on the patient’s symptoms
Source: PLoS One. 2022 Mar 15;17(3):e0265230. doi: 10.1371/journal.pone.0265230 (PMC8923505; doi:10.1371/journal.pone.0265230)
Supplement: S5 Table — (PDF) [file pone.0265230.s005.pdf]

S5 Table. Diagnoses and symptoms observed in 35 cases with encephalitis/meningitis etc.

Diagnosis	Symptoms
Encephalitis	Dehydration, disturbed consciousness, speech loss
Acute necrotizing encephalopathy	Disturbed consciousness, mental state abnormal, dyspnoea, Na decreased, focal seizures*, disorientation*, speech arrest
Cerebellitis	Vertigo/lightheadedness
Acute encephalitis	Disturbed consciousness
Encephalitis	Convulsion, disturbed consciousness, somnolence/insomnia
Encephalitis autoimmune	Blood pressure decreased, disorientation*, lack of response to verbal stimulation, increased heart rate*, tachypnoea*
Encephalitis autoimmune	Hypogeusia, anorexia, tremor, difficulty walking*
Encephalitis autoimmune	Convulsion, disturbed consciousness
Encephalitis	Pyrexia/chills, disturbed consciousness
Diffuse encephalopathy	Convulsion, disturbed consciousness, somnolence/insomnia
Encephalitis	Pyrexia/chills, somnolence/insomnia, oculomotor nerve palsy, anorexia
Encephalitis	Convulsion, mental state abnormal, tremor
Disorder resembling cerebellar type Hashimoto's encephalopathy	Vertigo/lightheadedness, nausea/vomiting, tremor
Encephalitis autoimmune	Vertigo/lightheadedness, nausea/vomiting, anorexia, tremor
Longitudinally extensive transverse myelitis	Feeling of weakness/muscular weakness, pain, voiding symptoms
Longitudinally extensive transverse myelitis	Numbness, feeling of weakness/muscular weakness, voiding symptoms
Anti-GAD65-mediated limbic encephalitis	Feeling of weakness/muscular weakness, disturbed consciousness, memory impairment, attention disorder*, disorientation*, delayed recall*
Cerebellar ataxia type Hashimoto's encephalopathy	Vertigo/lightheadedness
Meningoencephalitis autoimmune	Malaise, pyrexia/chills, headache, mental state abnormal
Encephalitis autoimmune	Convulsion, disturbed consciousness
Myelitis	Feeling of weakness/muscular weakness, paralysis, voiding symptoms
Encephalitis autoimmune	Paralysis, dyslalia
Acute parencephalitis	Vertigo/lightheadedness, diplopia, dyslalia
Myelitis and polyradiculitis	Malaise, feeling of weakness/muscular weakness, vertigo/lightheadedness,

Diagnosis	Symptoms
	difficulty walking*
Myelitis transverse	Numbness
Suspected encephalitis autoimmune	Headache, vertigo/lightheadedness, nausea/vomiting
Limbic encephalitis	Malaise, pyrexia/chills, disturbed consciousness,
Immune-related acute cerebellitis	Vertigo/lightheadedness, diplopia, dysarthria
Limbic encephalitis	Feeling of weakness/muscular weakness, disturbed consciousness, dyspnoea, memory impairment
Encephalitis brain stem	Malaise, anxiety/depression, dysphagia
Encephalitis	Convulsion, visual field defects
Encephalitis autoimmune	Disturbed consciousness, mental state abnormal
Immune-mediated encephalitis (encephalitis/cerebritis)	Headache, eosinophil count increased
Cerebellar type Hashimoto's encephalopathy	Convulsion, stiffness, vertigo/lightheadedness, tremor
Immune-related encephalitis	Pyrexia/chills, disturbed consciousness, mental state abnormal

*Symptoms that were reported but are not specifically included in the options available in the application.
