# Supplementary material for: Validation of an online application to identify potential immune-related adverse events associated with immune checkpoint inhibitors based on the patient’s symptoms
Source: PLoS One. 2022 Mar 15;17(3):e0265230. doi: 10.1371/journal.pone.0265230 (PMC8923505; doi:10.1371/journal.pone.0265230)

S1 Fig. Uplift in sensitivity for predicting upper gastrointestinal disorders after incorporating links to common symptoms described in 19 cases retrieved by the literature search.

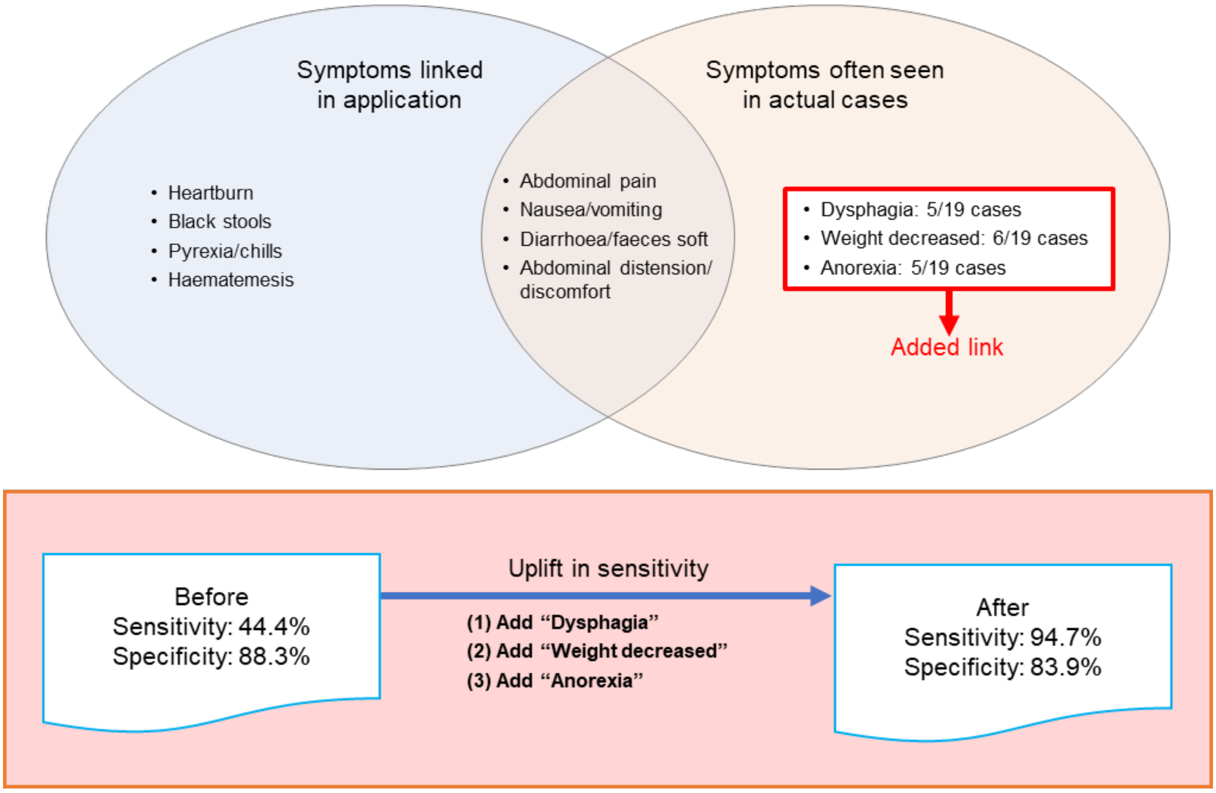

Supplement: S1 Fig — (PDF) [file pone.0265230.s006.pdf]
