# Supplementary figures and images for: Validation of an online application to identify potential immune-related adverse events associated with immune checkpoint inhibitors based on the patient’s symptoms
Source: PLoS One. 2022 Mar 15;17(3):e0265230. doi: 10.1371/journal.pone.0265230 (PMC8923505; doi:10.1371/journal.pone.0265230)

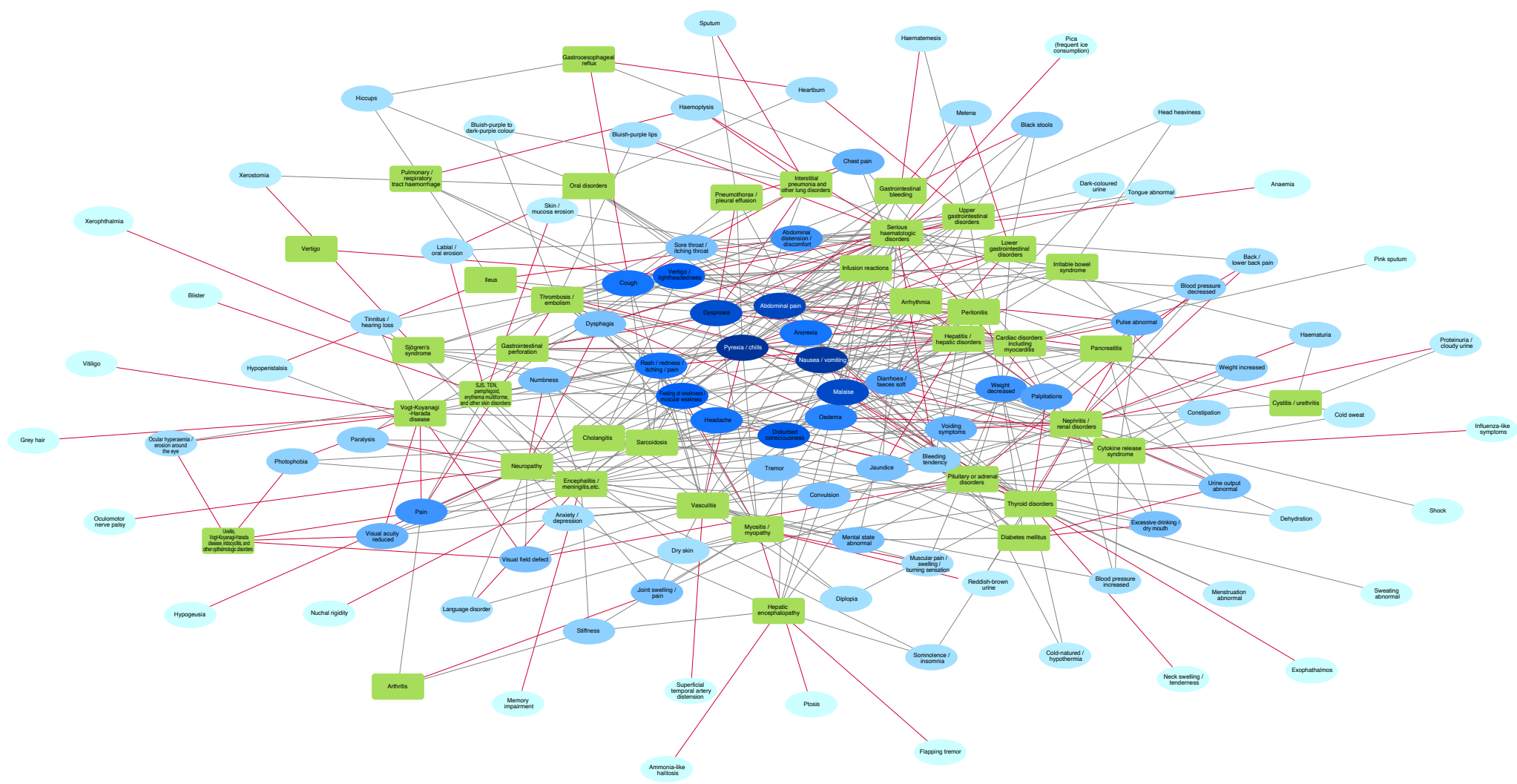

Supplement: S1 File — The symptoms and irAEs are linked using red or grey lines. Red lines indicate high specificity of a symptom for a particular irAE, grey lines indicate low specificity of a symptom to each linked irAE. Symptoms are colored blue, where the intensity of color represents the number of irAEs the symptom is connected to. Light blue symptoms are linked to one or a few irAEs, and are classified as highly specific symptoms. By contrast, dark blue (e.g., pyrexia/chills, nausea/vomiting, and abdominal pain) indicates broad symptoms, which are linked to many irAEs. (PDF) [file pone.0265230.s008.pdf]
